# Indianapolis Medical Society

**Published:** 1848-02

**Authors:** 


ARTICLE II.

### INDIANAPOLIS MEDICAL SOCIETY.

The Physicians of Indianapolis, Indiana, have organized a Medical Society, under the above name, which is to be composed of the regular practitioners of medicine of Marion and the adjacent counties.

Dr. J. H. Saunders, President; Dr. L. Dunlap, Vice President; Dr. J. S. Robbs, Recording Secretary; Dr. T. Bullard, Corresponding Secretary; and, Drs. G. Mead, C. Parry, and L. Dunlap, Censors. Its meetings will be held on the evening of the first Saturday of every month.

We are glad to see the profession organizing in different parts of the country. It shows a spirit of improvement, and cannot have other than a good effect, when a desire to advance in knowledge and improve in social intercourse is the object of association. E

---

ARTICLE III.

